# Secondary Subtrochanteric Fracture After Atypical Femoral Shaft Fracture Treated With Intramedullary Nail

**DOI:** 10.7759/cureus.17544

**Published:** 2021-08-29

**Authors:** Young-Ho Cho, Jeong Duk Suh

**Affiliations:** 1 Orthopedic Surgery, Daegu Fatima Hospital, Daegu, KOR; 2 Orthopedics, Daegu Fatima Hospital, Daegu, KOR

**Keywords:** subtrochanter fracture, secondary fracture, atypical femoral fracture, diaphisis, intramedullary nail

## Abstract

Intramedullary (IM) nail fixation is widely used for the treatment of atypical fractures of the femoral shaft. The configuration and location of proximal interlocking screws are unique to each nailing system and maybe transverse or oblique in direction. The authors experienced two cases of incomplete secondary fractures at the subtrochanteric region after IM nail fixation for atypical femoral shaft fractures. The proximal screw fixation of the two cases was different from one another. One was fixed with a spiral blade plus transverse screw and the other was fixed using an oblique direction screw from the greater trochanter to the femoral neck base. Based on our experience, we recommend only using a proximal locking screw toward the head when using an IM nail for the treatment of atypical femoral diaphyseal fractures.

An 82-year-old female patient who had been fixed with an IM nail for the treatment of atypical femoral shaft fracture 13 months ago visited the outpatient clinic with pain in the right hip joint for one month. Local hot uptake was observed at the proximal interlocking screw insertion site around the subtrochanteric region on bone scan. A simple removal of the proximal locking screw was enough to treat the incomplete fracture.

A 79-year-old woman visited the emergency room for pain in the right hip joint. On the radiograph, the right femur was found to be fixed with an IM nail, and an incomplete fracture line around the lower border of the lesser trochanter was observed. This patient was treated by replacing the IM nail with a reconstruction nail.

When using an IM nail for the treatment of atypical femoral shaft fractures, it is appropriate to insert only the screw toward the femoral head for proximal fixation to prevent secondary subtrochanteric fracture.

## Introduction

The diagnosis and treatment of atypical femoral fractures (AFFs) have been scarcely reported in the literature, and they are believed to be associated with long-term bisphosphonate drug use [1-3]. Intramedullary (IM) nail fixation is known to be the standard treatment, but the incidence of delayed union and nonunion is relatively high, ranging from 12.9% to 31.2%, and the need for additional surgery such as plate fixation with or without bone graft and exchange nailing has also been reported [1-7].

We have come across two reports of secondary fractures in the subtrochanteric region after IM nail fixation of atypical femoral shaft fractures in the literature [8,9]. The authors experienced two cases of incomplete secondary subtrochanteric fractures after IM nail fixation for diaphyseal AFF, and we discuss them in this report along with a review of the literature.

This study was approved by the Institutional Review Board at Daegu Fatima Hospital (IRB No. DFE21ORIO092), which waived the need for informed consent.

## Case presentation

Case 1

**Figure 1:**
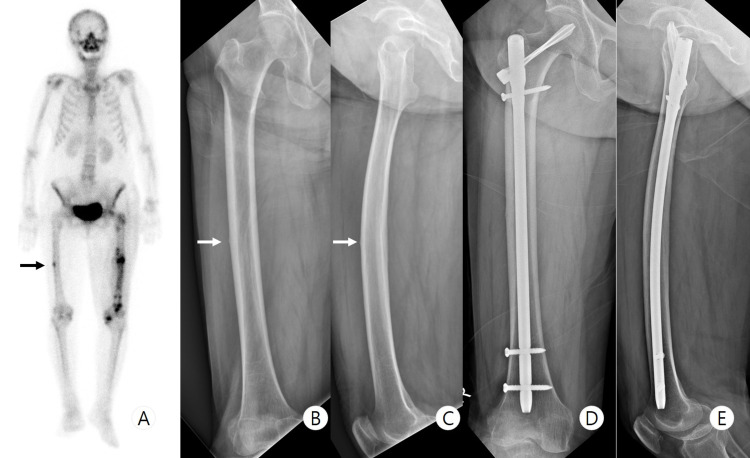
Previous preoperative imaging and six-month follow-up X-ray - case 1 Preoperative bone scan (A) shows hot uptake at the right femur midshaft. White arrow indicates cortical hypertrophy and radiolucent line on preoperative anteroposterior (B) and lateral (C) radiographs. The six-month follow-up X-ray images show the disappearance of the radiolucent lint and the decrease in cortical hypertrophy (D, E)

An 82-year-old female patient visited the outpatient clinic with pain in the right hip joint for one month. There was no trauma such as falls, and the right femur had been fixed with a cephalomedullary nail due to an incomplete atypical fracture of the right femur shaft 13 months ago and the bone union had been achieved (Figure 1).

**Figure 2:**
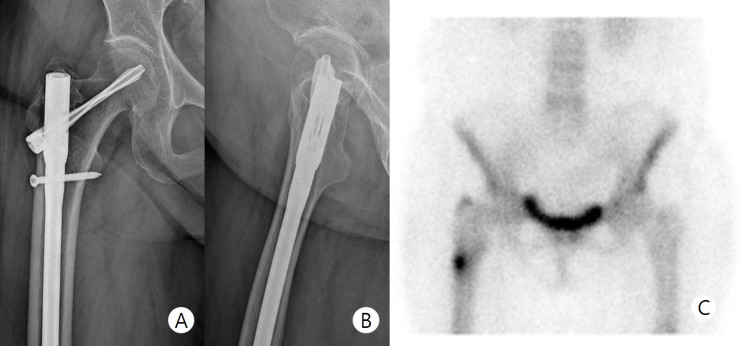
Preoperative imaging - case 1 Preoperative anteroposterior (A) and lateral (B) radiographs show no specific finding. Bone scan (C) shows hot uptake at lateral femoral cortex at the subtrochanteric region

Her blood test revealed that 25-OH vitamin D3, osteocalcin, and collagen type I C-telopeptide (CTX) were 20.3 ng/mg, 4.78 ng/mL, and 0.197 ng/mL, respectively. She had a history of taking bisphosphonates (risedronate) for 40 months before the onset of the atypical fracture. She had stopped taking the drug after the fracture and had been given denosumab three months before the onset of right hip pain after the bone union was completed. The T-score of the spine was -2.8. No specific findings were observed on radiography, and local hot uptake was observed at the proximal interlocking screw insertion site around the subtrochanteric region on bone scan (Figure 2). There were no local signs of infection such as erythema and swelling. Blood tests to check for infection, such as a complete blood count (CBC), erythrocyte sedimentation rate (ESR), and C-reactive protein (CRP), were in the normal range. Despite one month of conservative treatment including non-weight bearing, the patient complained of pain and constantly limping gait while walking.

**Figure 3:**
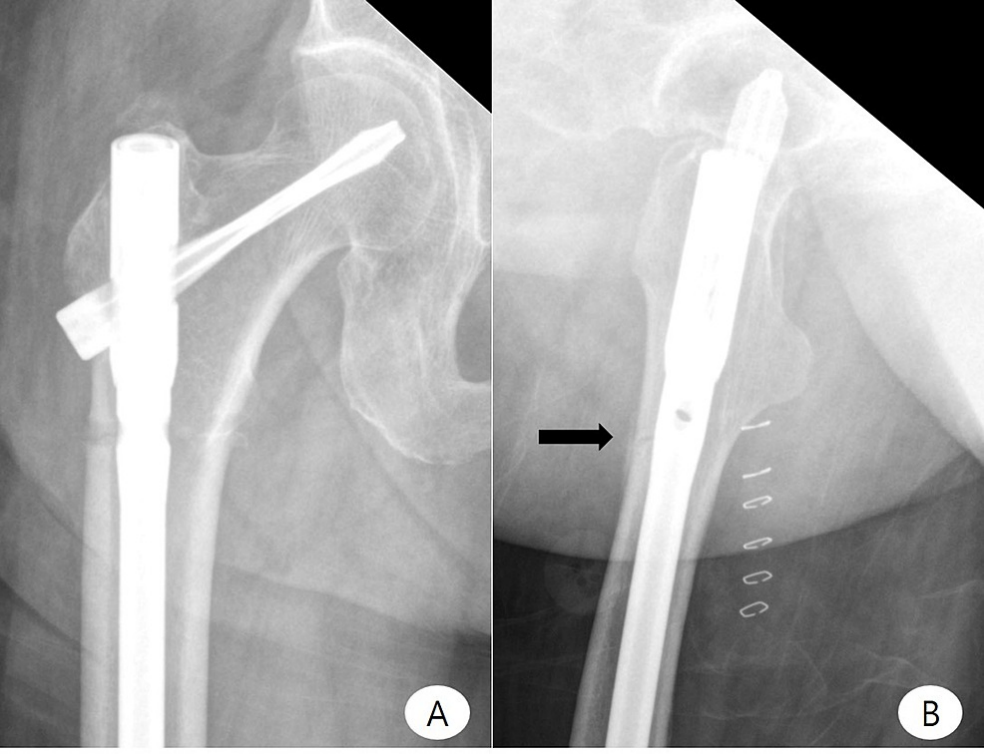
Postoperative imaging - case 1 Postoperative anteroposterior (A) and lateral (B) radiographs. Black arrow indicates cortical thickening and incomplete fracture line at the anterior femoral cortex. The images show complete healing of the previous incomplete fracture at the subtrochanteric region

The surgical treatment was considered, and the proximal interlocking screw inserted in the transverse direction was removed. In the postoperative lateral radiograph, an incomplete fracture line was identified in the anterior cortex where the screw was removed (Figure 3).

**Figure 4:**
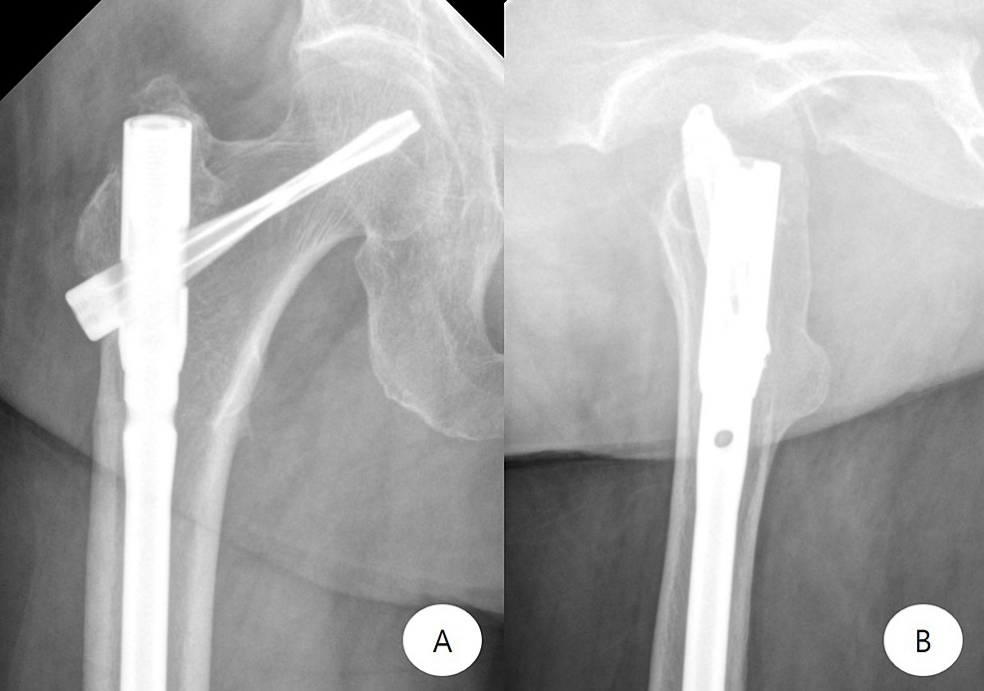
Follow-up imaging - case 1 Thirteen-month follow-up anteroposterior (A) and lateral (B) radiographs show complete healing of the previous incomplete fracture at the subtrochanteric region, incomplete fracture line, and anterior cortical thickening at the lower border of the lesser trochanter

After the operation, calcium and vitamin D supplements were administered, and the patient was able to walk without pain and any aid like a walker and cane in two months. A bone defect at the interlocking screw insertion site eventually consolidated, and the fracture line disappeared in six months (Figure 4).

Case 2

**Figure 5:**
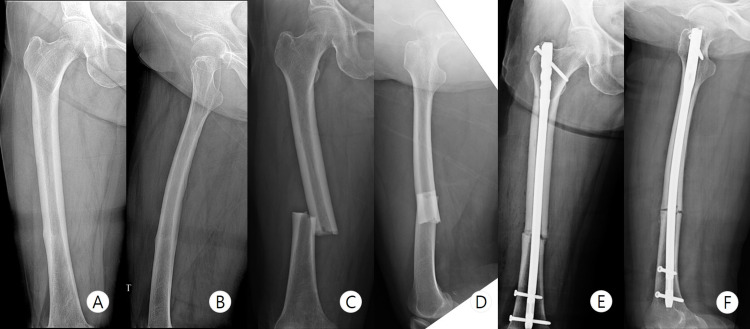
Previous imaging - case 2 Right femur anteroposterior (A) and lateral (B) radiographs 10 months before the complete fracture show lateral cortical hypertrophy, and the radiolucent line indicates incomplete fracture. Complete fracture of the right femur (C, D) and immediate postoperative X-ray (E, F)

A 79-year-old woman visited the emergency room due to pain in the right hip joint. There was no history of trauma. An IM nail fixation had been performed at another hospital for a complete atypical femoral shaft fracture of the right femur 81 months ago (Figure 5). The old fracture was found completely united. She had been taking bisphosphonates for three years before the fracture of the femoral shaft, which she had stopped after the fracture surgery, and had been taking it again for the past one year. The range of motion of the right hip and knee joint was normal on physical examination, but the weight-bearing was not possible. Blood tests showed that 25-OH vitamin D3, osteocalcin, and CTX were 20.8 ng/mg, 7.84 ng/mL, and 0.199 ng/mL, respectively. The T-score of the lumbar spine was -2.7.

**Figure 6:**
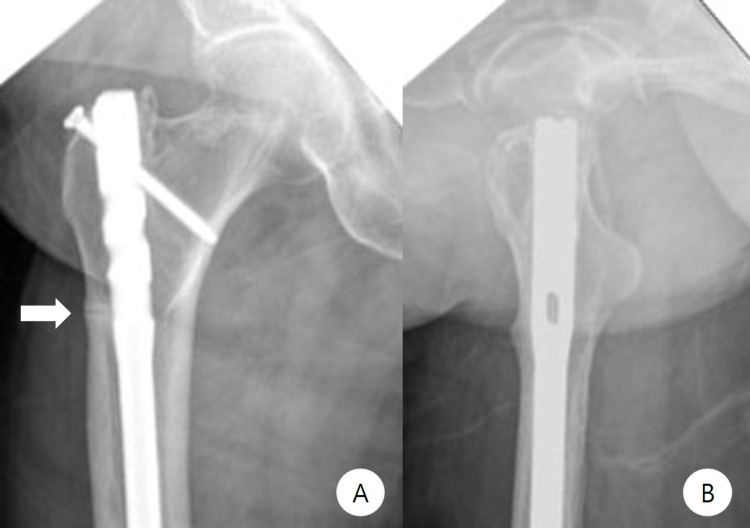
Preoperative imaging - case 2 Preoperative anteroposterior (A) and lateral (B) radiographs show incomplete fracture line (white arrow) and anterior cortical thickening at the lower border of the lesser trochanter. The images show complete healing of the previous fracture

The radiographs taken immediately after the first operation had confirmed that the femur had been perforated in the lesser trochanter area for insertion of the proximal transverse interlocking screw, but the screw had not been inserted. On the current radiograph, the right femur was fixed with an IM nail, and radiolucent lines and cortical thickening on anteroposterior and lateral views were found around the lower border of the lesser trochanter (Figure 6). It was diagnosed as an incomplete subtrochanteric femoral fracture.

**Figure 7:**
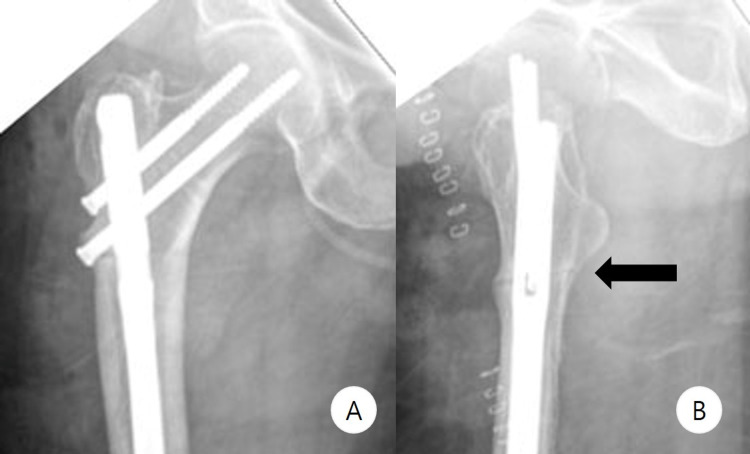
Postoperative imaging - case 2 Postoperative anteroposterior (A) and lateral (B) radiographs show complete fracture line (black arrow) after the reconstruction nail fixation

**Figure 8:**
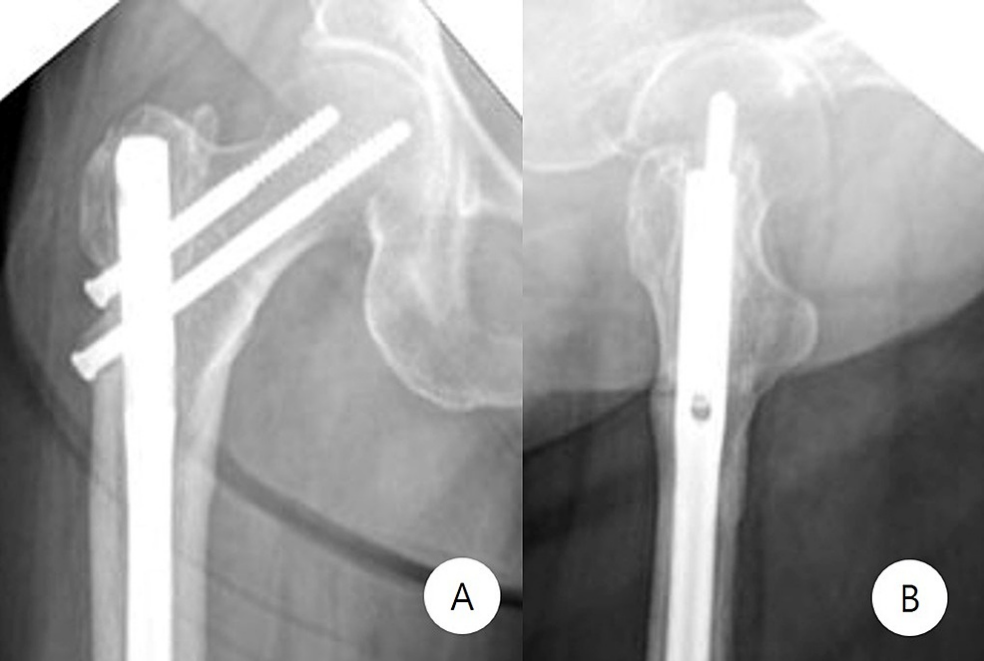
Follow-up imaging - case 2 Six-month follow-up anteroposterior (A) and lateral (B) radiographs show complete healing of the subtrochanteric fracture

The surgical treatment was considered. We removed the inserted IM nail and refixed using a cephalomedullary nail (Figure 7). The incomplete fracture progressed to complete fracture during the surgery, but the displacement of the fracture did not occur. After six months, bone union was confirmed on the radiograph (Figure 8), and the patient was able to walk without pain.

## Discussion

AFFs occur in the subtrochanter or shaft with absent or minor trauma. For the surgical treatment of these atypical femur fractures, the preferred method is IM nail fixation, which promotes secondary bone healing [1-4].

Several studies have reported a high frequency of delayed union and nonunion as postoperative complications of AFFs. Weil et al. [7] reported that 46% of patients who developed AFFs after using bisphosphonate drugs for a long period of time and underwent surgical treatment with IM nails required additional surgery due to delayed union and nonunion. Egol et al. [10] reported that it took about 8.3 months to obtain complete bone union after surgical treatment with an IM nail in a patient with AFFs that had occurred after taking bisphosphonates for more than five years.

In addition, there have been reports of complications related to IM nails in AFFs. Bonifacio and Syson [11] have reported the breakage of an IM nail after fixation for an AFF. As per this report, it should be noted that even if an IM nail is used for AFFs, the possibility of delayed union or nonunion is high and this result can lead to nail breakage.

Fang et al. have reported a case of a metal breakage that occurred in the proximal part 30 months after the fixation of the nail for an AFF in the femoral shaft. They recommended the use of a solid nail that is more rigid than a slotted nail [7]. Kim et al. have also recommended that if IM nails are selected for fixation of atypical fractures in the femoral shaft, screws directed toward the head and neck should be inserted for proximal fixation, if possible [8].

In case 1, a prophylactic IM nail fixation had been performed for an incomplete right femoral shaft fracture, and 13 months after the operation, the patient complained of pain in the right proximal thigh without a history of trauma. An incomplete fracture was identified at the transverse screw insertion site around the subtrochanteric region. This fracture was thought to be caused by the stress concentration in the subtrochanteric area, as the stress applied to the femoral shaft disappeared with IM nail fixation. Since the blade heading to the head and neck was already inserted, only the screw inserted in the stress concentration area was removed without any further treatment. It was expected that new bone would be made in the hole where the screw was removed and the fracture would heal, and it subsequently did.

In case 2, IM nail fixation had been performed for an atypical complete fracture in the femoral shaft previously and the proximal interlocking screw had been fixed at the trochanteric region. A fracture line was found at the lateral cortex of the lower border of the lesser trochanter 81 months after the operation. It was expected that a new reconstruction nail could overcome stress concentration on the lateral cortex and the fracture would heal.

An important point in the mechanism of AFFs is the tensile force applied to the femur. In the case of femurs with anterolateral bowing, fracture occurs in the shaft with the apex of the bowing, and in the femur with little or no bowing, the maximum tensile force is applied to the lateral cortex of the subtrochanteric region. As described above, when micro-damages accumulate in the area to which the maximum tensile force is applied and the rate at which the damage heals does not reach the rate of healing, it is expected that a fracture will be clinically presented on a radiograph with pain [12].

In patients with femoral shaft fracture, the tensile force applied to the shaft is decreased after fixing the IM nail. Even if tensile force is applied, it does not proceed to a clinical fracture due to the protection by an IM nail. It is believed that similar forces can be applied to the subtrochanteric area, and fractures may occur in this area if the stress concentration area is not properly protected. In the first case, though the blade toward the head of the femur was inserted, there was room for the stress concentration due to the insertion of a screw in the transverse direction around the lesser trochanter. In the second case, the screw heading to the head of the neck was not inserted, and hence the tensile force applied to the lateral cortex around the lesser trochanter could not be protected by the inserted IM nail.

## Conclusions

When using IM nail for the treatment of atypical femoral shaft fracture, it is appropriate to insert only the screw toward the femoral head for proximal fixation to prevent secondary subtrochanteric fracture.
